# Supplementary material for: Salivary Oxytocin Concentration Changes during a Group Drumming Intervention for Maltreated School Children
Source: Brain Sci. 2017 Nov 16;7(11):152. doi: 10.3390/brainsci7110152 (PMC5704159; doi:10.3390/brainsci7110152)
Supplement: Supplementary file 1 [file brainsci-07-00152-s001.pdf]

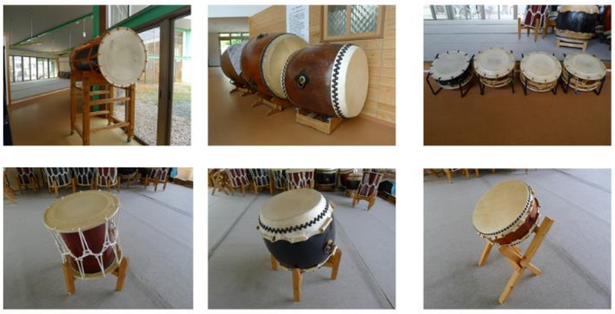

Supplementary Figure S1. Various types of Wataiko (Japanese percussions) used for music intervention in group.

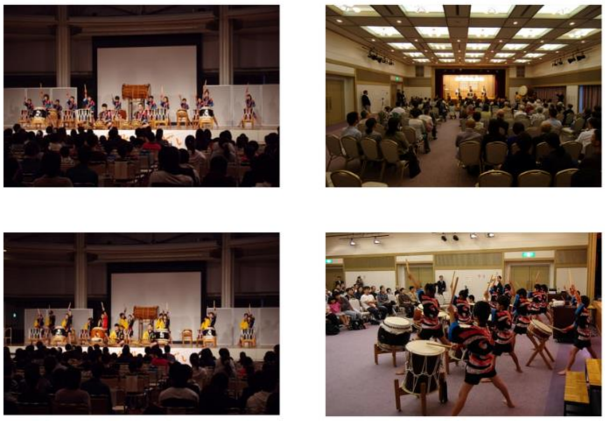

Supplementary Figure S2. Recitals performed in front of audiences in various stages in wearing with a festival uniform

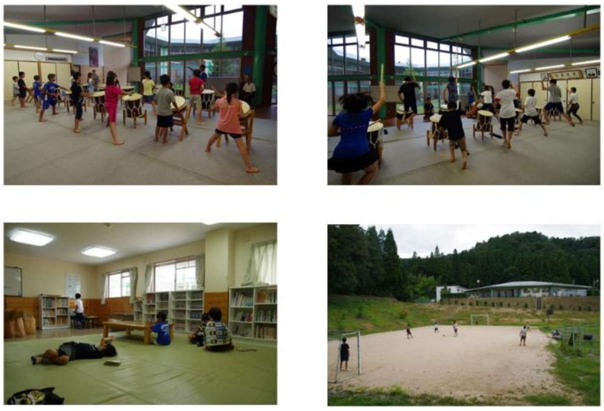

Supplementary Figure S3. Upper panels, Practice in a hall. Lowe panels, Free sessions.

Recital / Free session	Higher than average	Lower than average	Average
Higher than average	1 2 1 → restress autistic ADHD	1 → gentle	2 → obedient
Lower than average	1 1 → nervous	7 4 2 → well-behaved quiet no self-confidence	
Average			1 3 1 → feeble apprehension

Supplementary Figure S4. Oxytocin concentrations of individuals before recital and free play sessions. Number indicates elementary school boys in black, junior high school boys in blue and elementary school girls in red. Characteristics of individuals taken from teachers' records are shown.

Recital / Free session	Higher than average	Lower than average	Average
Higher than average	3 1 2 → hyperactive	1 1 → obedient dull tic	1 2 1
Lower than average	1 → Asperger		2 2 → well-behaved
Average	1	2	3 3 1

Supplementary Figure S5. Changes in oxytocin concentrations of individuals before and after recital and free play sessions. Number indicates elementary school boys in black, junior high school boys in blue and elementary school girls in red. Characteristics of individuals taken from teachers' records are shown.
